# Experiences Relating to Sexual Well-Being Among Muslim Gynecological Cancer Survivors: A Systematic Review of Qualitative Studies

**DOI:** 10.1089/whr.2023.0105

**Published:** 2024-06-27

**Authors:** Samaneh Alinejad Mofrad, Heidi Green, Shailendra Sawleshwarkar, Ibrahim Alananzeh, Ritin Fernandez

**Affiliations:** ^1^ Department of Nursing and Midwifery, School of Nursing, Mashhad University of Medical Sciences, Mashhad, Iran.; ^2^ Australian Centre for Health Engagement, Evidence and Values (ACHEEV), School of Health and Society, University of Wollongong, Wollongong, Australia.; ^3^ Faculty of Medicine and Health, Sydney Infectious Diseases Institute, The University of Sydney, Westmead, Australia.; ^4^ University of Wollongong Dubai, School of Humanities, Social Sciences and Health, Dubai, United Arab Emirates.; ^5^ School of Nursing and Midwifery, University of Newcastle, Callaghan, Australia.; ^6^ Centre for Transformative Nursing, Midwifery, and Health Research: A JBI Affiliate Centre.

**Keywords:** gynecological cancer, Muslim, sexuality, sexual wellbeing, women

## Abstract

**Background:**

Gynecological cancers are one of the most important threats to women's health worldwide. The objective of this review is to synthesize and present the best available evidence on the experiences relating to sexual well-being among Muslim women with gynecological cancer.

**Methods:**

The databases searched included Web of Science, Scopus, SID, Google Scholar, ProQuest, MEDLINE, and CINAHL from the inception of the database until August 2021. The review was guided by the JBI methodology used for qualitative systematic reviews. Findings were collated using the meta-aggregation method through JBI SUMARI.

**Results:**

Eight studies involving Muslim women cancer survivors were included in the review. Meta-synthesis of the eight included studies generated 59 findings, which were organized into 14 categories and combined into four synthesized findings.

**Conclusions:**

Gynecological cancer and its treatment results in numerous challenges with sexual well-being among Muslim women cancer survivors. Providing information about sexual activity following gynecological cancer, better communication from health care professionals, and support from the husband is essential to overcome the struggle with intimacy and femininity experienced by the women, thus improving the sexual quality of life of Muslim gynecological cancer survivors.

## Background

Gynecological cancers are the one of most important threats to women's health worldwide^1^ and can produce a negative effect on the multidimensional concept of sexuality in women.^2^ First, physical changes due to treatments after the diagnosis such as hysterectomy can negatively affect women's sexuality.^3^ Further, physical changes, including scar formation in the vagina, shortened vagina,^4^ vaginal atrophy,^5^ vaginal dryness,^4,6,7^ and dyspareunia,^4,5^ can lead to inactivity and dysfunction in sexual relations.^7–9^

Moreover, sexual inactivity can also be due to decreased sexual interest, fear of cancer recurrence,^7^ and fear of infection.^8^ Second, treatment for gynecological cancers has psychological and social effects on these women. In a qualitative study,^4^ psychosocial and interpersonal experiences of women with these cancers were reported to interact with each other, and so, their sexual relationships were affected. The most common experienced difficulties include anxiety about being rejected by their husbands, altered womanhood and sexual desire, and changes in interpersonal relationships.^4^

On the other hand, research involving Muslim women with these cancers highlights that sexuality remains a sensitive topic within this demographic, often regarded as a taboo that is not openly discussed.^8,10^ Consequently, these women may not receive sufficient information about sexuality due to the societal discomfort in discussing it openly.^3,4,8,10^ Muslim women believe that having sexual relations with their husbands is a religious obligation, and avoidance of it is considered as a sin.^11^

Consequently, they feel compelled to maintain their sexual relationships regardless of the circumstances. Thus, it is essential to recognize the significance of addressing sexuality as a pivotal aspect of the treatment process for Muslim women with gynecological cancer, on par with other medical considerations.^12^ Consequently, it is important to be aware of the perceived notions of sexuality among Muslim women receiving nursing care for gynecological cancers. Hence, the aim of this qualitative review is to explore the experiences relating to sexual well-being among Muslim women with gynecological cancer.

## Materials and Methods

This systematic review was guided by the JBI methodology used for qualitative systematic reviews.^13^ A protocol has been published in PROSPERO (CRD42021289527).

### Search strategy

The three-step search strategy that was conducted in August 2021 sought to locate both published and unpublished qualitative studies. Databases were searched from inception until August 2021 and only included studies published in the English language. An initial limited search was undertaken using MEDLINE; following this, keywords that were contained within the title, abstract, and MESH terms were identified within the relevant studies to inform the structured search strategy.

Using the developed search strategy, Web of Science, Scopus, SID, Google Scholar, ProQuest, MEDLINE, PsycINFO, and CINAHL were searched.

The keywords used in the database search were: (Gynecological cancer OR Genital Cancer OR Reproductive System OR Genital Neoplasms OR Uterine Cervical Neoplasms OR Uterine cancer OR Cervical cancer OR Ovarian cancer OR Ovarian Neoplasms OR Neoplasm, Ovary OR Ovary Cancer OR Cervical Neoplasm, Uterine OR Cervix Neoplasm OR Cervix Cancer OR Vulva cancer OR Vulval cancer OR Vulvar cancer OR Endometrial cancer OR Corpus cancer OR Cervical Carcinoma OR Vaginal cancer) AND (Islam OR Islamic OR Muslim OR Muslims OR Mohammedanism OR Arab*) AND (Sexuality OR sexual health OR sexual well-being OR sexual Life OR Sexual Behavior OR Sexual Dysfunction OR Sexual Activity OR Sex Behavior OR Behavior, Sexual OR Sexual experience* OR Sexual intercourse OR Sexual problem* OR Sexual pleasure OR Intimacy OR Sexual issue* OR Sexual concern* OR Sexual function* OR Sexual intimacy).

A structured search for unpublished studies included Health and Medical Collection and Theses (including the Nursing and Allied Health Database), ProQuest Dissertations, and WorldCat. The final step in the search strategy included the authors reviewing the reference lists of all the selected studies to identify any additional studies to be incorporated into this review.

### Inclusion and exclusion criteria

Qualitative studies that included Muslim women aged 18 years and older with gynecological cancers were considered for inclusion in this review. Studies were included if they reported the experiences of Muslim women with gynecological cancer and their sexuality. Studies were excluded if they (1) reported on aspects of gynecological cancer but did not refer to sexuality, (2) discussed the husband's experience of women with gynecological cancer, and (3) were conducted using quantitative methods.

### Data extraction and synthesis

Following the assessment of each selected study to match the inclusion criteria, data were extracted from the included papers, with the assistance of the JBI System for the Unified Assessment, and Review of Information (JBI SUMARI) data extraction tool.^14^ The detailed data extracted from each of the studies included participant characteristics and the sample size, study design, experiences of sexual difficulty, and strategies that were used by patients.

The quotes (illustrations) from participants in the included qualitative studies (qualitative results) were extracted verbatim with the inclusion of a women's quote to support the meaning of the results. The JBI levels of credibility^14^ were used to rate the evidence: as credible, unequivocal, or unsupported. An unequivocal ranking demonstrates that the evidence is realistic and is not open to challenge; a credible ranking indicates that the evidence is convincing, but could be contested; and an unsupported ranking refers to the evidence that is not consistent with the findings.^14^

Qualitative data were collected through the meta-aggregation approach, involving the compilation of findings at the verbatim subtheme level from individual papers. This method acknowledges the potential presence of common findings among the reviewed publications. Subsequently, the process entailed grouping findings with similar meanings to establish categories.

These categories were constructed by grouping at least two analogous findings within each category. The resulting categories were amalgamated to produce synthesized findings. Each synthesized finding was accompanied by an explanatory statement encapsulating the collective meaning of a group of conceptually similar categories.

#### Methodological quality assessment

Methodological quality assessment was undertaken using the JBI qualitative studies critical appraisal tool.^15^ The critical appraisal for each study was conducted by one reviewer (S.A.M.) and checked by a second reviewer (R.F., I.A., or H.G.). Each standard was given a score (Yes = 2, No = 0, Unclear = 1), providing a total score of 20 for each paper. Each total score was then converted to a percentage, with only studies scoring at least 70% included in the review. Any disagreements between reviewers were resolved via discussion, third reviewer, or author.

## Results

### Search results

The search conceded a total of 546 citations, of which 282 were duplicates. The remaining 264 citations were screened for relevance using the title and abstract, and 58 were retrieved for potential inclusion. The references of these papers were scrutinized for potential inclusion; however, no new papers were identified. Sixteen full-text papers were assessed for eligibility. Four of the 16 studies did not meet the inclusion criteria during reading of the full text and were excluded, and four of the remaining 12 studies were excluded because they did not meet critical appraisal criteria.

**FIG. 1.**
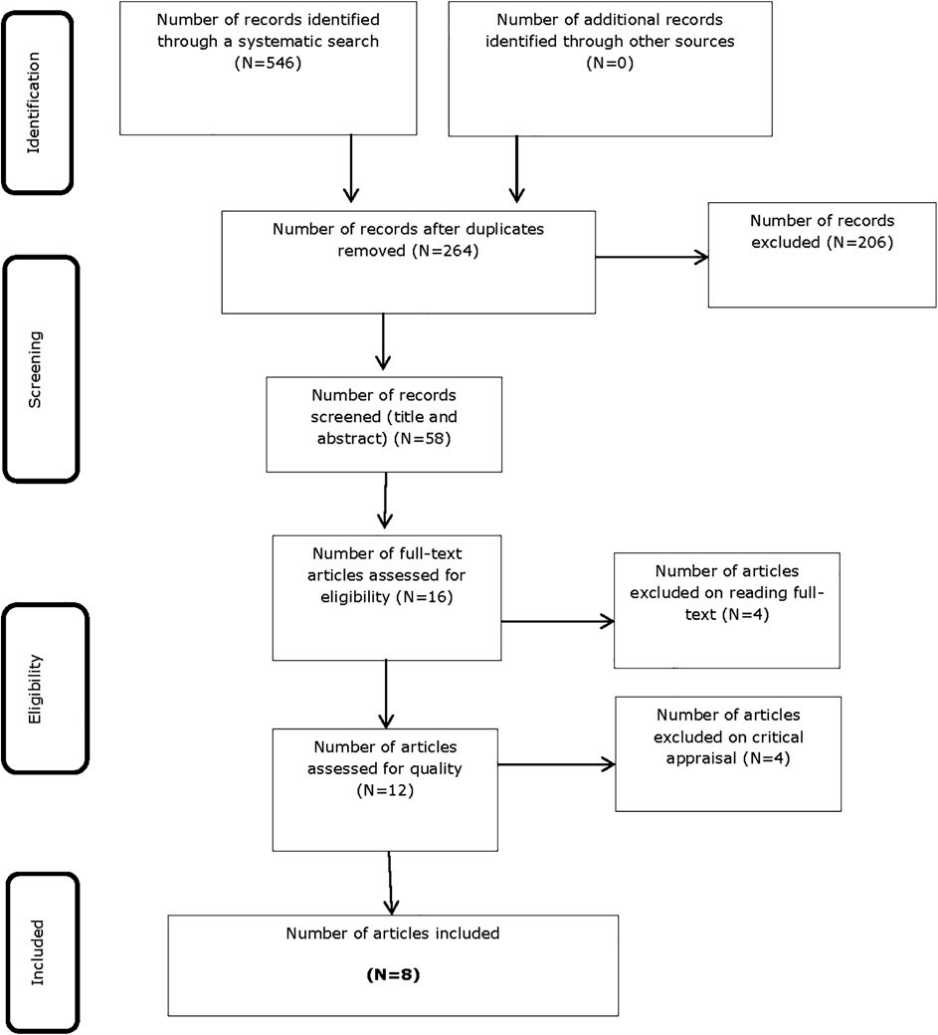
PRISMA flow diagram. From Moher et al.^61^

Reasons for exclusion included: (1) the study did not relate to women's sexuality following gynecological cancer; (2) not in the English language; (3) explored the husband's experience of women's sexuality after a mastectomy; and (4) study did not report on qualitative methods. A total of eight studies were included in the final systematic review (Fig. 1).

### Methodological quality

All eight studies were critically appraised for methodology quality based on the JBI critical appraisal checklist for qualitative studies. No studies were excluded based on methodological quality, as all scored higher than 70%. The highest score was 90% [15, 24], and the lowest score was 80% [25–30]. There were no disagreements between reviewers regarding the critical appraisal results. The critical appraisal results for the included studies are outlined in Table 1.

**Table 1.** Critical Appraisal

Citation	Criteria										Results (%)
	Q1	Q2	Q3	Q4	Q5	Q6	Q7	Q8	Q9	Q10	
Yaman and Ayaz^16^	y	y	y	y	y	N	Y	U	Y	Y	17/20 (85%)
Serçekuş et al.^17^	Y	Y	Y	Y	Y	N	Y	Y	Y	Y	18/20 (90%)
Reis et al.^10^	Y	Y	Y	Y	Y	N	Y	Y	N	Y	16/20 (80%)
Alinejad Mofrad et al.^20^	Y	Y	Y	Y	Y	N	Y	Y	Y	Y	18/20 (90%)
Kebede et al.^62^	Y	Y	Y	Y	Y	N	Y	Y	Y	Y	18/20 (90%)
Birhanu et al.^63^	Y	Y	Y	Y	Y	N	N	Y	Y	Y	16/20 (80%)
Akyüz et al.^64^	Y	Y	Y	Y	Y	N	Y	Y	Y	Y	18/20 (90%)
Afiyanti et al.^45^	Y	Y	Y	Y	Y	N	Y	Y	Y	Y	18/20 (90%)
%	100.0	100.0	100.0	100.0	100.0	00.0	87.0	87.0	87.0	100.0	

### Characteristics of included studies

Narratives from the eight qualitative studies involving 184 women aged between 31 and 65 years were included in the review (Table 2). Studies were published between 2008 [29] and 2021 [25], with four studies conducted in Turkey, two studies in Ethiopia, one in Indonesia, and one in Iran. The studies were conducted in various hospital settings and gynecology and chemotherapy center clinics. Seven studies used semi-structured interviews for data collection, and one study used focus groups. Characteristics of the included studies are outlined in Table 2.

**Table 2.** Characteristics of Included Studies - Interpretive and Critical Research Form

Study	Methods for data collection and analysis	Country	Phenomena of interest	Setting/context/culture	Participant characteristics and sample size	Description of main results
Afiyanti et al.^45^	Semi-structured interviews	Indonesia	Experiences of sexual relationship after a nurse led psychosexual intervention	Outpatients of the Cipto Mangunkusumo National Referral Hospital in Jakarta.	16 couples (16 females, 16 males) Mean age 43.12 (females); 47.45 (Males)	Four themes emerged: (1) lessening suffering; (2) supporting each other to regain sexual life, finding reasons to do or undo sexual intercourse (2a), “How can I bring back my wife's sexual desire?” (2b), “Finally I get to a climax” (2c); (3) becoming more intimate and caring about each other, and (4) “Now I'm getting more confident”
Reis et al.^10^	Semi-structured interviews	Turkey	To explore the sexual problems of Turkish cancer patients with gynecological cancer	Hospital setting	30 females Age range 28–68 years	Four themes emerged: (1) Body image (2) Gender role (3) Sexual functioning (4) Reproductive ability
Mofrad et al.^11,41^	Face-to-face semi-structured interviews Conventional content analysis	Iran	To reveal what sexuality life difficulties Iranian women with gynecological cancers experience	Brachytherapy ward in Imam Reza Oncology Radiotherapy Center and Women's Clinic and Cancer Department of Ghaem Hospital of Mashhad City.	Sixteen Iranian women with gynecological cancer	Three themes emerged from the data: (1) participant's struggle to maintain the sexual monopoly of the husband, (2) deterioration of intimacy, and (3) unpleasant bed-life experiences.
Kebede et al.^62^	Face-to-face semi-structured interviews Conventional content analysis	Ethiopia	To explore the psychosocial experiences and the needs of women diagnosed with cervical cancer.	Black Lion Specialized Referral Hospital (BLSRH), Addis Ababa	A total of 12 women (patients) and key informants (staff members).	Three themes emerged from the data: (1) The first theme is on socio-demographic profiles; (2) the second is devoted to presenting the diagnosis processes and the women's reactions toward the results; (3) the third presents quality of life after diagnosis; and the final theme is about the needs of the women who participated in the study.
Birhanu et al.^63^	Thematic analysis of focus group discussions using theoretical framework of the Health Belief Model (HBM).	Ethiopia	To describe the perceptions of men, women, and community leaders regarding awareness, and treatment-seeking behaviors for women with symptom or signs of cervical cancer in Ethiopia	Random selection of parents from Addis Ababa and Jimma zone in Ethiopia randomly selected from a district, then village and school (one urban and one rural) where girls were called for HPV vaccination.	A total of 168 participants - 112 males 56 females Age range of 21–70 years	Findings showed four thematic groups: (1) awareness of CC (2) perceived etiology of CC (3) perceived benefits of treatment (4) perceived barriers to treatment:
Akyüz et al.^64^	Descriptive phenomenology using semi-structured interview	Turkey.	To describe the meaning of the gynecologic cancer experience from the perspective of Turkish women	Gynecologic oncology outpatient clinic of the Gulhane Military Medical Academy in Ankara, Turkey.	31 participants 19 women with gynecologic cancer Age range 43–70 years	Seven themes: (1) experiences during the diagnosis period (2) experiences during the treatment period (3) the effect of cancer on family life (d4) changes in daily life: limitation on social activities (e5) coping methods and support sources (f6) the meaning of illness (g7) experiencing the illness as a woman
Yaman and Ayaz^16^	Semi-structured, in-depth question	Turkey	Psychosocial problems faced by women in Turkey during their illness with gynecological cancer, and how they cope with these problems	Attending gynecology service for chemotherapy	17 women Mean age 54.8	The psychological problems found included: (1) frustration and despair, (2) depression, (3) inability to control anger, (4) disruption in body image, and (5) problems with their sex lives.
Serçekuş et al.^17^	Semi-structured interviews and analyzed by using a content analysis method	Turkey	To reveal what sexuality difficulties Muslim women with gynecological cancers experience and how` they overcome them.	Oncology center of a university hospital	18 females Age range 28–68 years	Three major categories: (1) situations that make sexual life difficult, (2) impact of cancer on sexual life, and (3) coping

### Synthesized results

Meta-synthesis of the eight included studies generated 59 findings (subthemes verbatim from individual studies), which were organized into 14 categories (based on similarity of meaning) and combined into four synthesized findings (explanatory statements) (Table 3).

**Table 3.** Narratives from the Eight Qualitative Studies

Synthesized findings	Categories	Findings	Illustrations
Reluctance to engage in sexual activity due to physical and emotional affect, fear of rejection by the husband, and lack of sexual information from health professionals	Reluctance to engage in sexual activity	Refrained from sexual intercourse	“No sexual intercourse for 5 years. How can a cancer patient have sexual intercourse? A wound with blood like a river. How can you have sexual intercourse?”^16^
		Sexuality became less important	“I don't want to have sexual intercourse. My womb is like this (pointing to her colostomy bag); I can't have it when I have this bag. How can I get into sexual contact if the waste material is flowing into the bag? My sutures can be separated. They have already been separated in the same place 7 times, and then they have been closed. Therefore, we haven't had sexual intercourse for 3 years. (64 years old, endometrial cancer”^17^
		Did not consider lack of sexual intercourse as a problem because of their ages	“My husband and I don't have any problems with sexuality. We are old. (59 years old, endometrial cancer)”^17^
		Spouses were not attracted to them anymore	It [sexual intercourse] never takes place. … Never, for sure. … I have wounds everywhere, how could it happen? He sees the wounds and he doesn't want it.”^16^
		Running away from intercourse	: “After hysterectomy, I feel no sexual arousal… whenever my husband want to take me to the bedroom, I shout … I have no feeling for this…when he wants to have an intercourse, I become very angry …”(P18)^20^
	Lack of sexual information and communication impacting sexual activity	Not given information about effects of cancer on sexuality	“I am having sexual reluctance. I do not know if this is normal because I do not get any information from the health staff. (21 years old, ovarian cancer)”^17^
		Not receive any information about allow for having sexual relation or not	“I'm not having sexual intercourse. I don't know if I can have it. Nobody told me anything about it. I didn't ask anyone about it. (64 years old, ovarian cancer)” ^17^
		Expected to be given information about them by health professionals	“I wish the doctor, or the nurse had informed me. The questions in my mind could have disappeared. .I couldn't ask any questions so that they shouldn't think I was planning to have sexual intercourse. (50 years old, cervical cancer)”^17^
		Could not talk to health professionals about sexuality because she was not married	“Since I was not married, I couldn't talk to my doctor about sexuality. The other health staff didn't give any information to meI No one in the society is talking about sexuality. (21 years old, ovarian cancer)”^17^
	Pain after and during sexual activity	Pain after sexual intercourse and decreased vaginal lubrication	“‘You can't have sexual intercourse for 5–6 months due to pain You feel such severe pain as if your vagina was cut with a knife.^17^
		Intense pain they felt during sex lessened their sexual desire	“don't want it as I used to. Toere is dryness and the pain is too much. My husband continually talks about this matter; he says ‘you were not like that before’. Before the disease, sometimes 1 started sex, and sometimes he did. it did not matter at all. Nowadays my husband asks ‘What is going on? lfl don't initiate, you don't even remember’. Because refrain, my husband feels upset.’ (A 50-year-old woman with endometrial cancer)” Page 143
		Aches and pains felt during sexual intercourse	“I feel a!ot of pain and burning there. it f'eels like there is a wound there. which is incised with a knife. A pain like that. Not the entrance pan, but the inside of it aches, especially when his penis is deep inside. 1 am always preoccupied with that pain. fear that I will feel the same pain every time, and this fear discourages me from the desire and will to be with my husband. (A 43-year-old woman with cervical cancer)”^10^
		Avoided sexual relations in the 6 months after treatment believing	I was scared for the first 5–6 months. Just in case. But I felt that my surgical wounds had not healed completely. So I did not want it much. Then we started doing it slowly but I had pain, especially at the back. We did not have sex for awhile and my partner understood. We don't have any problems now (Participant 17, cervical carcinoma, 57 years old). Page 244 Akyüz et al. (2008)
	Emotional rejection by the husband	Rejected by her husband because of cancer	Although I was his first wife, my husband got married to another woman. Currently I am not sleeping with my husband. Of course, I do not have any sexual feeling. Although I do not have any sex feelings, I feel that I am rejected.” Page 9 Kebede et al. (2016)
		Could not remember having any romantic time with their husbands since their diagnosis	It is 1 year since I stopped sex.…my doctor suggested I can have sex depending on my health condition. Sometimes, I give signs of sexual desire to my husband. However, his response is ‘do not worry about sex’. I believe what my husband wishes is to see my health condition improve Page 9 Kebede et al. (2017)
	Fear of husband's rejection during sexual intercourse	Scars on the operation area may affect their sex life	‘I already have a big operative scar on my abdomen. 1 also put on a [lot of weight after the operation. 1 am sometimes obsessed by the thought that my husband does not find me as beautiful and attractive as he used to …’ (A 30-year-old woman with ovarian cancer)” ^10^
		Anxious about the possible reactions of their husbands	This man will certainly want some things from me. But what will he do with me after that? Will he look after me all my life as if caring fora blind woman? thought like that.’ (A 60-year-old woman with cervical cancer)” ^10^
		The idea that the cancer could be transmitted to the partners through sexual relations	‘We had not had sex for 1 year. 1 feared that I could transmit my illness to my husband. 1 always escaped from him. Whenever he wanted to have sex with me, 1 made up different excuses. I said had pain, I did not feel well, etc. But soon realized that it could not go on like that, so I told him the reason. Then he said, ‘it does not matter, what will happen after all? What if I had the same illness? Would you escape from me as well?’ (A 48-yearold woman with ovarian cancer)”^10^
	Husband's reaction to his wife's hysterectomy	CC is cause of divorce as husbands do not want to live with a woman who has CC	“… men ignore their wives if they have symptoms of CC and the victim women themselves feel self enacted stigma and feel themselves as less important person and divorce will happen” Page 5 Birhanu et al. (2012)
		Her husband felt very sad since she did not give him a child	‘My husband is the only son of his parents. 1 strongly believe that he wishes he could have a son of his own blood to maintain his family lineage. Tough he says nothing to me about this matter, he surely dreams of it.’ (A 30-year-old woman with ovarian cancer)”^10^
		Decrease a sense of femininity to his wife after hysterectomy	My husband and I are became emotionally distant after the cancer started … but the worst part was when the doctor asked my husband for permission to remove my uterus.. … he thinks that if I have my uterus removed, I will no longer be a woman … That's why he does not caress me at all … and he does not sleep next to me … …” (p17) ^16^
Struggle with intimacy and sexual pleasure due to body image changes, loss of femininity, and sexual anxiety	Lack of sexual pleasure during intercourse	Spouses would not get any pleasure from it	“I have had no [sexual] relations for two years since I got diagnosed. … I hold off from my husband as they [sexual organs] all are gone. … Can I have sexual intercourse with my husband? Can he have pleasure?” ^16^
		Vaginal sutures had negative impact on their sexuality	“I want to avoid sexual intercourse for a while since I have pain. I won't have it for at least 1 year since there are sutures inside my womb. I have had a very important operation.”^17^
		Sexual intercourse just to make husband happy	“I can't have pleasure. I can say I'm experiencing unwillingness. I'm having sexual intercourse just to make my husband happy without being willing to do it. (33 years old, ovarian cancer) “^17^
		Decreased capacity to reach orgasm	“I rarely have an orgasm. Let us say that we have sex three times a week, only in one of them I have an orgasm, or I do not have one at all.’ (A 44-year-old woman with cancer)”^10^
		Incomplete penis penetration due to radical hysterectomy	‘My vagina had shortened; there was dryness as well. We could not have intercourse, after each time we tried, my husband felt had, he got frustrated and angry 1 was having therapy, the therapist wanted my husband to participate as well, but he did not come. Finally he was persuaded. (A 45-year-old woman with cervical cancer)”^10^
		Feeling sexual intercourse	“.…My husband does not understand me at all … A few days ago he forced me to have sexual intercourse, I told him I wasn't feeling well … but he said that “this is your problem” .… I have to have sexual relation with my husband … It is not important for him that I am satisfied from sexual intercourse or not”…”(P17) ^20^
	Unpleasant feeling due to their altered body image Fear and anxiety to engage in sexual activity	Experienced disruption in their body image	“I used to go out, take care of myself, buy clothes. Does it suit me? Does that suit me? I used to have my hair done. Nothing suits me now. I have an abdominal hernia here. No hair, no eyebrows. I buy nothing for myself. If I do, it is only for necessity. ”^16^
		Body image reflects a psychological experience	“I cannot go to the bathroom alone. … I don't know why. … Do I fear? Is it because I am bald? I cannot look at a mirror. … Maybe I will go to the bathroom easier if my hair gets better.”^16^
		Being disturbed by the excessive weight she has put on after the treatment	‘How I have put on weight I have reached 80 kilos. My husband says it does not matter for him. and that I am beautiful anyway. But who knows how he really fee)s. I don't even like myself. Why would he? So I feel clumsy.’ (A woman aged 59 with ovarian cancer” ^10^
		Physical difficulties such as pain, nausea, vomiting, insomnia, fatigue, and hair loss	The main difficulty was during chemotherapy. Losing my hair affected me a lot. I did not want to look in the mirror after I lost my hair. It was as if I was looking at someone else (Participant 13, ovarian carcinoma, 58 years old). Page 243 Aky üz et al. (2008)
		Losing their fertility and not being able to have children created a great trauma	‘I would probably not feel so sad because of the fact that my uterus was removed if I had at least one child.’ (A 28-year-old woman with ovarian cancer)”^10^
		Decrease the feminine identity and cause her to part from her fertile friends	You are marked Yes, marked l have two marks, one, you have cancer and the other; you are infertile. It is sad difficult to go on living like that You cannot be among people easily, you hesitate, and you become shy.’^10^
		A woman without a uterus is not a woman	“If a woman is denuded of her reproductive organs, she tums into an empty sack. This really is the case for me as well … 1 feel like an old and empty sad.1 feel my stomach is empty and barren like a wasteland.’ (A 55-year-old woman with endometrial cancer)” ^10^
		Fear of sexual intercourse	“There are sutures in the vagina. I do not want to harm my vagina. (64 years old, ovarian cancer)” ^17^
		Women feared that the disease could get worse recur, and spread	“I don't want to have sexual intercourse. I'm scared. The disease spread to my liver after 3 years. I fear that the disease can spread to other parts of my body. (56 years old, vulvar cancer)”^17^
		Anxiety about the effect of seminal fluid on her disease	‘I should say, as we are talking woman to woman. that my husband has never let his semen fluid into me ever since the operation, believing that the fluid might make my disease worse and cause it to recur.’ (A 29-year-old woman with ovarian cancer)”^10^
		Connected to their psychological situation	‘it changes from time to time; when I am frustrated. 1 don't feel anything. lf I am ok, 1 can have an orgasm’ (A 32-year-old woman with ovarian and endometrial cancer)”^10^
		Anxiety before and during sexual intercourse	“I feel that my vagina is closed through the treatments, and there is no cure for it…my husband feels pain when he wants to have internal sex… then he could not have successful intercourse and therefore he becomes upset… and starts to complain… this situation is so stressful for me and it becomes an unpleasant sexual experience .…” (P13) ^20^
Religious and cultural obligation to fulfill the sexual needs of the husband	Meeting the sexual needs of the husband is religiously obligatory	Making their spouses happy as a religious duty	“I don't want to have sexual intercourse, actually. I have difficulty in it. I tell myself I should satisfy his wish. It is also a sin not to fulfill it. (71 years old, ovarian cancer)’ Page 4
		Forcing herself to meet husband's sexual needs to avoid sin	If I don't serve my husband, I am afraid of getting sin … So now my desire starts to come up. Page 299^45^
	Trying to accommodate the husband's sexual needs is culturally necessary	Persuade herself to allow his husband for short-term marriage	: …Therefore, I decided to find a temporary (Sigha) wife for my husband. It was important for me to find a modesty woman. Finally, I found a woman and they had a short-term marriage for one night. In the morning, I gave an extra money to her to make sure that she would be available next time…” (P20)^20^
		Concern about unmet husband's sexual need	“….Although I was banned from sexual intercourse from the physician, But I continued it, until I bled severely once or twice. When I told the doctor, she told me that it was a big mistake. But you know, my husband is a man and has sexual needs. Still, we didn't have sexual relations during the over the past month and now he (husband) has realized that This situation will continue. I feel compassion for him..” (participant No.9) ^20^
Psychosexual interventions and husband's support assisting to improve sexual satisfaction	Benefit from psychosexual interventions	Medications/cream to cope with physical symptoms affecting their sexual life	“I felt I had dryness. I had never experienced it. I told about it to my doctor. The doctor prescribed medications. After I got them, I felt comfortable. (64 years old, ovarian cancer)””^17^
		Psychosexual intervention helped them manage with pain during intercourse	“Well, basically I do what I have been told. I need to get relaxed first, then I'll start. When I began having an intercourse after chemo, I could not do it. I had so much pain as it was dry … it was painful … but now it is much better.” Page 299^45^
		To have sexual intervention with lack of orgasm and satisfaction	…maybe I'm also near menopause so my sexual interest is not coming back yet.” Page 299^45^
		They were able to reach orgasm again, even though it was difficult for them	” Now my husband helps me to get ready, as you have taught us. Previously, I could not reach a climax, but then I practiced what you taught – the Kegel [pelvic floor] exercise, finally, I get to a climax.” Page 300^45^
		Different ways that worked for them to help achieve the orgasm, including changing the sexual position	“Now I can get to a climax. With my husband's help, I can finally have a climax, even more my husband – he seems to be unaffected by my illness. But well, we cannot have a climax at the same time. I have it sooner then he would” Page 300^45^
		Enjoyed more intimacy and caring about each other	“I feel more intimate to him. Before having sex, he would tell me to take shower, to pass water, and he would do it too … wow, plenty of rituals. But it makes me happy, I feel that he's getting more attentive to me.” Page 301^45^
		Couples also learned to communicate more openly to each other	“After we had the session with you, now my husband would always talk or ask me how I am doing everyday. Before he went off to work, he would remind me to eat well. Before we have sex, he would also check with me if I'm ready or not … It wasn't like that before you came to us. I'm also improving. Now I would let him know if I'm not ready yet. So we communi cate to each other. I used to have no idea what to communicate before.” Page 301^45^
		Self-esteem also grew from the knowledge they obtained in the nurse-led psychosexual intervention	“The lessons you gave me helped clear out my anxiety that having sexual intercourse can make me die … my friends said that. But now, I am just confident to do it.” Page 302^45^
	Husband's support lead to have better sexual experiences	Sufficient support from their spouses	“Naturally, my sexual desire decreased after my womb was taken out. However, when you love somebody, you endure everything. My husband is very considerate. When we didn't have sexual intercourse, he did not consider it as a problem. (55 years old, endometrial cancer)” Page 4
		Noticing positive sides of their status made them relaxed	“I thought I was lucky because I experienced the disease when I was single. If I was married, the disease could cause difficulties in sexuality. I've always had to see its positive aspects. This is very important. (21 years old, ovarian cancer)”^17^
		The help from their husbands did work to improve their sexual desire	After my husband helped me I could get relaxed. I could feel ready and have the desire… but it took time indeed.” Page 300^45^

#### Synthesized finding

Reluctance to engage in sexual activity due to physical and emotional affect, fear of rejection by the husband, and lack of sexual information from health professionals.

This synthesized finding encompasses six categories:

##### (1) Reluctance to engage in sexual activity

For Muslim gynecological cancer survivors, one of their main concerns was regarding engaging in sexual activity.^4,10,11^ Some women refrained from sexual intercourse because of consistent vaginal bleeding.^16^ Sexuality also became less important for some women while being treated for cancer, and others did not consider the lack of sexual intercourse as a problem because of their older age.^17^ Some women expressed that their spouses were not attracted to them, due to the lack of sexual activity and acceptance of the physical aspects of their cancer.^16^ Some women avoided intercourse because they had no sexual desire and got angry when their husbands wanted to have sexual intercourse.^11^

##### (2) Lack of sexual information and communication impacting sexual activity

The lack of sexual information provided was a challenging issue for many Muslim women, particularly experiencing a lack of communication about the impact on their sexual activity. In this regard, Muslim women's problems included not being given information about the effects of cancer on sexuality, not receiving any information on engaging in sexual relations while having cancer and expected to receive information from health professionals.^17^ Some unmarried women expressed their reluctance to discuss their sexuality and sex life with health care professionals because of their unmarried status.^17^ This hesitation is rooted in the belief prevalent in Muslim cultures that discussions about sexual needs should be reserved for married individuals, as engaging in sexual activity outside of marriage is considered unacceptable.^17^

##### (3) Pain after and during sexual activity

Experiencing pain during and following sexual intercourse was another sexual problem for Muslim women with gynecological cancer. In this regard, decreased vaginal lubrication and experiencing severe pain in their vagina as if it was cut with a knife were commonly reported.^4,14,15^ Also, intense pain they felt during sex lessened their sexual desire for future sexual activity (10). However, other women avoided sexual relations in the 6 months after treatment because they feared pain.^9,11^

##### (4) Emotional rejection by the husband

Some women experienced emotional rejection from their husbands because of their cancer.^7,11,12^ However, other women stated that their husbands got married to another woman after their cancer diagnosis, leaving women feeling rejected by their husbands.^11^ Further, some women could not remember having any romantic time with their husbands since their diagnosis, which was often between 6 and 12 months.^4,6^

##### (5) Fear of husband's rejection during sexual intercourse

One of the most important sexual problems in Muslim women with gynecological cancer was fear of their husband's rejection during sexual intercourse. This feeling was due to the physical scars in the surgical area and the anxiousness regarding the possible reactions of their husbands due to the lack of sexual intercourse related to their vaginal issues.^4,11,14^ Further, one interesting finding was the idea that the cancer could be transmitted to the husband through sexual relations. Some women were reluctant to engage in sexual activity due to this concern.^4,11,14^

##### (6) Husband's reaction to his wife's hysterectomy

Having a hysterectomy is a very cultural and social issue in Muslim countries. The concept of the uterus is a vital part of the woman's body, providing them with a sense of femininity and motherhood. Removal of the uterus due to gynecological cancer is very challenging for couples.^8,11^ Muslim women's husbands feel a sense of despair when they are not able to reproduce and provide them with a child, and for the women, this decreases their sense of femininity.^8,11^

#### Synthesized finding

Struggle with intimacy and sexual pleasure due to body image changes, loss of femininity, and sexual anxiety.

This synthesized finding incorporates four categories:

##### (1) Lack of sexual pleasure during intercourse

Lack of sexual pleasure was another issue that Muslim women with gynecological cancer reported. The common reasons for the lack of sexual pleasure expressed by Muslim women were that the vaginal sutures had a negative impact on their sexuality and caused severe pain during intercourse.^8,11,12^ Women also engaged in sexual intercourse to please their husband and not for their own pleasure.

Muslim women believe that meeting the sexual needs of their husbands is their religious duty, and therefore would have sinned if they did not oblige.^8,11,12^ Another reason for the lack of sexual pleasure during intercourse was the decrease in women's capacity to reach orgasm and stated that they lost the ability to reach orgasm.^8,11,12^ Incomplete penis penetration due to radical hysterectomy was another reason for lack of sexual pleasure.

Radical hysterectomy had shortened the vagina, making it difficult for their husbands to achieve deep penetration.^7,8,11,12^ Another issue for Muslim women involved a reduced desire to engage in sexual intercourse, leading to instances where their husbands pressured them into engaging in sexual activity when they were not willing or ready for it.^11^

##### (2) Unpleasant feeling due to their altered body image

Significant changes to a Muslin women's body image due to cancer treatments were another problem creating unpleasant feeling for them.^4,11^ Many women experienced a disruption in their body image, with unpleasant psychological events and experiences leading to this problem.^16^ Women were disturbed by the excessive weight they had put on after the treatment and this along with physical difficulties such as pain, nausea, vomiting, insomnia, fatigue, and hair loss resulted in changes in Muslim women's body image.^4,11^

##### (3) Hysterectomy means the end of femininity

One of the most significant challenges for Muslim women with gynecological cancer was the loss of femininity following a hysterectomy.^4,11^ The uterus has great significance for Muslim women, with a hysterectomy meaning they also lose their fertility, with the inability to have children creating trauma and distress. Further, a decrease in Muslim women's feminine identity can cause her to part from their fertile friends.^4,11,15^ Also, a woman without a uterus is not a woman who was the worth, was also an issue that was reported.^10^

##### (4) Fear and anxiety to engage in sexual activity

Another challenging issue for Muslim women with gynecological cancer was the fear and anxiety associated with sexual intercourse. Fear of engaging was often expressed by Muslim women to be associated with the feeling that the vagina will be harmed.^4,11,15^ Women also feared that the disease could get worse, recur, and spread.^4,6,11,15^ Further, anxiety was about the effect of seminal fluid on their disease and having anxiety before and during sexual intercourse.^6,9,15^

#### Synthesized finding

Religious and cultural obligation to fulfil the sexual needs of the husband.

This synthesized finding comprises two categories:

##### (1) Meeting the sexual needs of the husband is religiously obligatory

For Muslim women, meeting the sexual needs of their husbands is a very important issue that is related to their religious beliefs. Sexual activity makes Muslim women's spouses happy, and out of a sense of religious duty and to avoid sin, women force themselves to meet their husband's sexual needs.^8,11^

##### (2) Trying to accommodate the husband's sexual needs is culturally necessary

Strong bonds with their cultural beliefs meant that Muslim women are culturally obligated to accommodate their husband's sexual needs, despite being told by the health professionals to avoid sexual relations.^8,11^ Women experience cultural pressures, making them feel that they need to offer some alternatives to meet their husband's sexual needs, and in some instances, it could involve temporary marriage to another woman.

#### Synthesized finding

Psychosexual interventions and husband's support assisting to improve sexual satisfaction.

This synthesized finding encompasses two categories:

##### (1) Benefit from psychosexual interventions

Muslim women with gynecological cancer expressed experiencing unpleasant sexual relations.^1,9^ In some cases, Muslim women had painful sexual intercourse because of vaginal dryness and they could resolve it by using medications such as creams.^6,11^ Moreover, psychosexual interventions helped them manage with pain during intercourse.^10,14^ Some women were able to reach orgasm again, even though it was difficult for them.

Different strategies that worked for some Muslim women to help achieve orgasm included changing the sexual position.^3,11^ Couples also learned to communicate more openly with each other and enjoyed more intimacy through caring about each other.^3,4,14^ Finally, self-esteem also grew from the knowledge they obtained in the nurse-led psychosexual intervention.^3,4,14^

##### (2) Husband's support led to better sexual experiences

Support provided by Muslim women's husbands was a very important factor in experiencing pleasant sexual relations. Muslim women expressed that noticing the positive sides of their status made them relaxed; this, in addition to the help from their husbands, improved their sexual desire.^10,11,15^

## Discussion

This study demonstrates important issues in understanding Muslim women with gynecological cancer regarding their sexual problems and reveals how they connect and resolve these matters with their cultural and religious beliefs. Psychological difficulties that result from gynecological cancer not only have adverse effects on women's quality of life but also threaten women with a variety of challenges, especially regarding their sexual life.^2,5^

The first theme of our study was “Reluctance to engage in sexual activity due to physical and emotional affect, fear of rejection by the husband and lack of sexual information from health professionals.” Gynecological cancers lead to reluctance to engage in sexual activity for Muslim women due to situations that make a sexual relationship difficult. As the participants noted, it was not just the sexual relationships that were affected by the cancer, but also the intimate relationships were affected as well.^7,10–12^

Physical symptoms such as decreased vaginal lubrication and pain during sexual activity, menopausal issues, and fear of sexual intercourse were found to hurt sexual activities.^9,18^ The causes why the women experienced fear of sexual intercourse were that they thought their disease would recur, worsen, or spread to other organs of the body and could be transmitted to their spouses.^8,11^ Further, vaginal dryness and dyspareunia affected their sexual relations.^5,8,10,11^

A recent study on women with ovarian cancer reported that physical changes caused by cancer treatment were found to affect sexual intercourse and couple's intimacy.^19^ Other studies reported that diagnosis and treatments of gynecological cancers caused a lot of sexual challenges such as loss of sexual desire and problems with orgasm.^3,10,11^ The decrease in intimacy was also reported.^8,11^ Unpleasant experiences during sexual activity in couples after gynecological cancer are due to the physical and mental side effects of cancer and its treatments.^8,11^ Another result of this review was avoiding sexual relationships.^11^

The nervous reactions to any sexual request from their husband in women, paying no attention to the spouses' sexual needs, and reluctant to sleep with husband in the same bed were the problems reported by the women in some studies^7,8,11,12,20^ Trying to scare the husband from having sexual activity that can be related to a misconception about cancer and cancer treatment was one of the reasons for not having vaginal intercourse.^7,8,11,12^

For example, some women scared their husbands by indicating that the medicine or device in their vaginas could hurt them if they have vaginal intercourse.^11^ A recent study reported that Moroccan women tried to find excuses and ways to run away from having sexual intercourse after the diagnosis of cancer.^21^ The second theme of our study was “Struggle with intimacy and sexual pleasure due to body image changes, loss of femininity and sexual anxiety.”

The findings of this study show that Muslim women have concerns regarding their femininity, body image, and sexual relationships with their spouse following gynecological cancer.^8,10,11,22^ One study showed that, since gynecological cancers directly affect the female sexual organs, some women felt that they had lost their femininity after losing their cervix, ovaries, and uterus, and this led to emotional and sexual problems in their marital and sexual life.^11^

It should be noted that hysterectomy was an important subject that led to the reluctance of sexuality and therefore intimacy in couples.^23,24^ This had an adverse effect on intimate relationships.^25,26^ One study examined sexual experiences in women after a hysterectomy and showed a decrease in husband's intimacy due to negative feelings after the surgery.^27^

It is reported that another factor that led to decreased intimacy in Muslim women with gynecological cancer and their husbands is the fear of the impact of cancer on their fertility.^28,29^ This can be justified by the fact that fertility preservation has a fundamental importance for Muslim women and can affect their whole quality of life.^29,30^ The third theme of our study was “Religious and cultural obligation to fulfil the sexual needs of the husband.”

Although sexual dysfunction after gynecological cancer has been reported in the majority of studies,^31–33^ Muslim women's response to sexual challenges following gynecological cancer may be affected by several sociocultural issues and varies in the context in which they live.^5,21,34^ For example, gynecological cancer for Muslim women is considered fatal, especially in its impact on their marital and sexual lives^35,36^

This is because sexual satisfaction holds a significant role in the stability of marital relationships among Muslim women, and marital dissatisfaction stemming from sexual issues is a leading cause of marital discord and divorce in Muslim couples.^37–39^ It is worth noting that some Muslim women diagnosed with gynecological cancer engaged in sexual intercourse, even when they were not willing, due to their belief that fulfilling their spouses' sexual needs was their duty according to Islamic principles, and failing to do so would be considered a sin.^40,41^

Further, it was observed that many of these women regarded their sexual obedience as a manifestation of their heightened religious commitment.^42^ These results indicate that Muslim principles affect sexual behavior of Muslim women with gynecological cancer.^43^ The implications of sexual morbidities could be profound for Muslim women with gynecological cancer, ranging from enduring physical pain during sexual activity to domestic violence and divorce threat.^38,44^

Sexual relationships are deemed imperative for the Muslim wives due to strong patriarchal and religious culture in this context.^8,11,38,44^ All the participants in this review were Muslims: “A wife must not be reluctant to the husband's call for a sexual activity” is an Islamic teaching.^17,37^ However, many Muslims seem to pay no attention that Islam illustrates the conditions such as illness and menstruation, under which sexual intercourse cannot be imposed to a wife.^45^

Understanding the principles of Islamic teachings take a committed learning; many Muslims learn Islam instead by the conduct in the society.^46^ It should be noted that some husbands expressed their understanding of their wife's situation, so they restrained their sexual demands. Yet, most Muslim women in this review are seemingly bound to the former concept that sexual intercourse is a wife's main duty regardless of their circumstances.^8,11^

In fact, after the disease, women were concerned about meeting the sexual needs of their husbands; so at first they were trying to cover these needs inside the home.^47,48^ In a study, the experiences of sexual intercourse in Iranian women after menopausal surgery were reported,^49^ and the women were concerned about their husbands' sexual relationships with other women as they were not able to meet their sexual needs.^49^ It should be noted that some sexual behaviors, such as the liberty to choose sexual partners, are different in the various cultures.^50^ For instance, it is legal among Shias to have a temporary marriage. It means that men are allowed to have another wife, especially when the first wife is not able to fulfill their marital responsibilities.

But because of cultural barriers, temporary marriage is still unacceptable in most Muslims.^51^ This review revealed that the psychosexual interventions and the husband's support assisting to improving sexual satisfaction helped with improving the cancer survivor's sexual satisfaction.^11,45^ Studies also revealed that Muslim cancer survivors experiencing serious sexual challenges want to have a greater interest in accessing psychosexual information but yet they may not engage in such discussion unless it is started by the health care provider.^34,52^

Psychosexual interventions such as using medications to cope with physical symptoms affecting their sexual intercourse can help them to manage with pain during sexual activity.^53,54^ Further, most studies reported favoring results of the non-pharmacological interventions such as pelvic floor exercise, relaxation technique, and sexual counseling to manage vaginal symptoms.^55,56^ One study revealed that the couples seemed enthusiastic as they had participated in both the psychosexual interventions and the follow-up interview.^45^

As also suggested in some studies, open communication between couples is a main key to renegotiating sexual and intimate relationship.^56–58^ Improved sexual relationships in cancer survivors receiving specialist psychosexual programs were also found in previous studies.^56–58^ A consistent pattern was reported in a couple-based study in which the cancer survivors' partners adapted to the women's condition and had a perfect result in sexual function.^57^ The psychosexual programs helped the couples beyond their sexual relationships, including their sense of harmony as a couple, which is the goal of Muslim cultural value.^59,60^

## Conclusion

Women with gynecological cancers are not aware of the effects of these cancers on their sexual relationships, because in a Muslim context, sexuality seems to be taboo, so women may avoid asking questions about their sexual problems, and it can finally lead to the end of marital life in these women. Therefore, strategies to provide Muslim women with the opportunity to voice their sexual problems and create consultation and rehabilitation plans for survivors of gynecological cancer are urgently needed. Addressing these concerns and priorities among women may facilitate informed decisions and improve satisfaction and outcomes among couples.

## Study Strengths and Limitations

The strengths of the study include the use of the standardized JBI critical appraisal instrument for qualitative studies. Further, potential bias was decreased through the involvement of more than one reviewer. The validity is established by the recurrence of findings between studies. The use of the Meta aggregation approach enabled the categorization of each result reported in the studies without seeking to re-interpret the author's findings.

In addition, this approach allows for the development of generalizable statements.^15^ On the other hand, some limitations need to be noted. First, although a comprehensive search of the databases was performed, publications not indexed in these databases were not included in this study. In addition, this review only included studies published in English.

So, studies published in other languages could have been excluded. In addition, this review did not explore the potential variations in themes generated by publications from different countries. Future reviews should consider investigating this aspect.

## Abbreviations Used

None
